# The dual neural effects of oxytocin in autistic youth: results from a randomized trial

**DOI:** 10.1038/s41598-022-19524-7

**Published:** 2022-09-29

**Authors:** Adi Korisky, Abraham Goldstein, Ilanit Gordon

**Affiliations:** 1 The Gonda Multidisciplinary Brain Research Center, Bar-Ilan University, 5290002 Ramat Gan, Israel; 2 Department of Psychology, Bar-Ilan University, 5290002 Ramat Gan, Israel

**Keywords:** Neuroscience, Social neuroscience, Medical research, Psychology

## Abstract

Recent discoveries have highlighted the effects of oxytocin (OT) on social behavior and perception among autistic individuals. However, a gap persists in the literature regarding the potential effects of OT and the neural temporal dynamics due to OT administration. We explored the effect of OT on autistic individuals using magnetoencephalography (MEG), focusing on M100, M170, and M250, social perception-related components that tend to show atypical patterns in autistic individuals. Twenty-five autistic adolescents participated in this randomized, double-blind MEG study. Autistic individuals arrived at the lab twice and received an acute dose of intranasal OT or placebo in each session. During the scans, participants were asked to identify pictures of social and non-social stimuli. Additionally, 23 typically developing (TD) adolescents performed the same task in the MEG as a benchmark that allowed us to better characterize neural regions of interest and behavioral results for this age group in this task. A source-model beamformer analysis revealed that OT enhanced neural activity for social stimuli in frontal regions during M170. Additionally, in each of the preselected time windows, OT increased activation in the left hemisphere, regardless of the content of the presented stimuli. We suggest that OT increased the processing of social stimuli through two separate mechanisms. First, OT increased neural activity in a nonspecific manner, allowing increased allocation of attention toward the stimuli. Second, OT enhanced M170 activity in frontal regions only in response to social stimuli. These results reveal the temporal dynamics of the effects of OT on the early stages of social and non-social perception in autistic adolescents.

Trial registration: This study was a part of a project registered as clinical trial October 27th, 2021. ClinicalTrials.gov Identifier: NCT05096676.

## Introduction

Autism spectrum disorders (ASDs) are early-onset, highly prevalent neurodevelopmental disorders characterized mainly by impairments in social perception and interaction^1^, including deficits in processing emotional expressions, recognizing faces, and identifying social cues in various experimental paradigms ^2^^–^^4^. Although an increasing body of evidence indicates qualitative and quantitative differences in attention to social stimuli among autistic individuals, a new line of studies suggests that the reduced social perception that has been reported in the literature does not represent deficits in social perception but rather an alternative way of interpreting social cues that enables good social communication when interacting with each other^5^^–^^7^. These theories claim that the former explanations regarding social deficits are over-simplified and that social communication in autism is indeed different but not necessarily worst. This line of research highlights the need for a more precise examination of both early and late perceptual processes in autistic individuals while taking into account the high heterogeneity in this population. In the scope of social communication behavior modulation, one neuropeptide that has a crucial modulatory role is oxytocin (OT), a naturally occurring hormone produced in two hypothalamic nuclei in mammals ^8,9^. Various studies have shown that a single-dose of OT can modulate various social behaviors in autistic individuals, such as mentalization abilities, face perception, and social learning^10^^–^^16^. Despite these evidence, other studies have failed to replicate the social-related effects^16,17^ or suggest that they depend on several individual parameters and traits^16,18,19^ and thus it is still unclear who can benefit the most of it and what are the specific influences on the perception among autistic individuals. One interesting scientific direction in the context of OT and perception in ASD is its influence on the early stages of social perception and efficiency. While several imaging studies showed that OT administration modulates long terms neural activity within and between social-related brain regions^9,20^^–^^24^, most studies have not applied methods with high temporal resolution (such as electro- or magneto-encephalography) to investigate whether OT influence early neural perceptual efficiency during face and emotional expression perception. Some studies have shown that in typically developed (TD) individuals OT promotes face-sensitive neural responses by modulating of the N170 component^25^, a negative posterior neural component that counts to mark face encoding^26^. Other studies in the TD population show no effects of OT in early components during emotional face perception ^27^ or a modest effect on the late positive potential^28,29^ . Although the literature on the effects of acute OT during social perception in ASD is even more limited, studies have shown that a single dose of OT modulates late positive potentials depending on an individual’s anxiety and endogenous levels of OT^30,31^. Despite these encouraging results these few pieces of evidence mostly indicate that more study is needed regarding the early effects of OT on face processing in ASD to infer about possible potential therapeutic effects.

Therefore, this study aimed to investigate the effect of OT on early processes of social perception in autistic individuals. Namely, we wished to explore the effect of OT on M100 and M170, which have an important role in early attention toward visual perception and face processing^32^. and found to present atypical patterns in ASD ^33^^–^^37^. In addition, we aimed to inspect the effect of OT on M250, which a frontal reaction that emerges during face representation^35,36^ that is reduced in autistic individuals^37,38^. Based on previous studies showing that OT increases social-related neural responses^22,23,39^, our main hypothesis was that OT would increase the amplitudes of these three components in response to social stimuli.

In addition, using magnetoencephalography (MEG), which has both excellent temporal and spatial resolutions, we aim to explore the influences of OT specifically in social-related neural regions which, in the current study, found to differentiate social from non-social stimuli in the TD brain. We assumed that autistic individuals would show reduced neural activity in these regions and that OT would increase the activity of these regions to resemble more closely that of TD individuals.

Finally, while most of the studies mentioned above focused on the effects of OT on adults with and without ASD, we propose a study of the objectives in adolescence (age 12–18), a period where the ability to process others’ social signals is developing and essential, is important. Following recent electrophysiology studies that emphasize the neural differences in face processing networks between childhood, adolescence, and adulthood in ASD^40^, we chose to investigate the effect of OT on the developing brain of adolescents to enrich existing knowledge on the potential effect of OT on this age group.

## Methods and Materials

### Participants

After obtaining ethical approval (Declaration of Helsinki) from the Beer-Yaacov-Ness-Ziona Mental Health Center Ethics Board (#537/16), 32 adolescents with a diagnosis of ASD visited our lab at Bar-Ilan University twice. An additional study included a group of 26 typically developing (TD) which served as a benchmark for our task in the MEG. These participants visited the lab for one session only and did not receive any treatment, but only underwent the MEG procedure.

Four participants, two from the ASD OT study and two from the TD study, were unable to complete the session due to technical issues. Seven individuals, six from the ASD OT study group and one from the TD study, were excluded from the analysis due to a high percentage of artifacts, such as muscle movements. Thus, 24 autistic participants and 23 TD participants from both studies were included in the final analysis (See Figure S1 in supplementary information for CONSORT flow diagram).

Autistic participants for the OT study were recruited through "Bait Echad"—ASD clinical centers of the Association for Children at Risk and using social media ads. All participants in this group met the diagnostic and statistical manual of mental disorders (DSM-5) criteria for ASD, and clinical diagnoses of high-functioning ASD were confirmed using the Autism Diagnostic Observation Schedule (ADOS-2)^41^.

In the ASD OT study only, we also ensured that there was no comorbid intellectual impairment (cut-off was IQ > 80) using the Wechsler Abbreviated Scale of Intelligence (WASI)^42^.

All scores from the WASI reported in the article were based on a four-scale test and were normalized to standard T-scores (*M* = 50, SD = 10). The TD participants were recruited using online ads. In both studies, all participants were males, aged 12–18 years, were native Hebrew speakers and had a normal or corrected-to-normal vision (see Table 1 for demographic details of both groups). Before the experiment, all participants and their parents underwent a telephone screening interview regarding chronic medical problems, cardiovascular risk factors, CNS disease, other mental illnesses, and the use of prohibited medications (see supplementary information for a full approved medication list in the ASD study). In both studies, participants with other neurodevelopmental conditions, intellectual impairments, impaired vision, impaired hearing, a history of significant head injury or neurological illness, current substance dependence diagnosis, or metallic implants were excluded from the study. In the TD study, we did not conduct IQ and ADOS assessments, however, we exclude participants with medical, neurological, or social impairments based on the above-mentioned screening.

**Table 1:** Participant characteristics.

Variable	ASD OT study (*n* = 24)	TD study (*n* = 23)
Sex (M:F)	24:0	23:0
Age	*M* = 14.01 (1.63)	*M* = 15.08 (1.51)
Handedness (R:L)	19:5	20:4
WASI	*M* = 45.8 (8.6)	–
ADOS-2	*M* = 10.6 (2.5)	–

Note: WASI- Wechsler Intelligence Scale show subtest standardized T-score (*M* = 50, SD = 10), ADOS-2—Autism Diagnostic Observation Scale 2nd edition comparison score. No clinical or intellectual assessment was conducted in the TD study.

At the beginning of each lab visit, parents provided an informed consent form, and the participant provided verbal assent.

All participants were paid for their participation. Individuals in the ASD OT study received 500 NIS, and those in the TD study received 150 NIS.

### Procedure

Autistic adolescents participated in a randomized, double-blind placebo-controlled trial of OT effects. Participants in the ASD OT study visited the lab for two sessions (approximately one week apart). Based on a computer-generated list of random numbers, in one of the sessions, they received OT, and in the other, they received the placebo (PL). Intranasal doses of 40 international units of OT (IU)/mL were prepared by the "Maayan Haim" pharmacy, Israel. We used age-dependent dosing such that participants aged 13–18 years received a dose of 24 IU (3 puffs to each nostril), and younger participants (aged 12 years) received 16 IU. Forty–five minutes following intranasal administration^43,44^, participants underwent MEG scanning and were subjected to digital registration of the head position. General instructions were provided regarding the need to refrain from head or body movements. We ensured that all participants felt as comfortable and relaxed as possible during this period. Participants in the TD study underwent the same experimental procedure but did not receive OT or PL due to ethical constraints in Israel. Therefore, they do not constitute a control group for the ASD OT study. They only provide a benchmark for the MEG task, which allows us to better define task performance and neural regions of interest (ROIs).

### Paradigm

**Figure 1:**
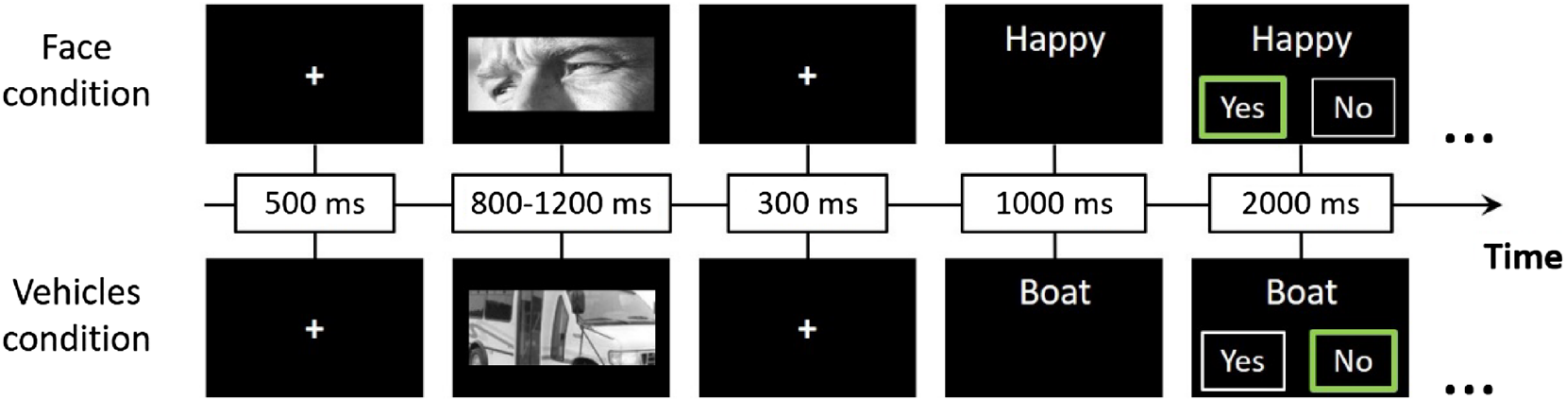
Behavioral paradigm—an example of two separate trials. The experimental procedure was adapted from the 'Reading the Mind in the Eyes' Task (RMET). It consisted of eight blocks—half contained pictures of faces (social condition, see example above the line) and the other half contained pictures of vehicles (non-social condition, see example below the line). The task lasted approximately 13 min in total. The order of the blocks and the order of the pictures inside each block were randomized and counterbalanced across participants. The three dots at the end of each row mark the continuity of the block.

The effect of OT on social perception was assessed using a well-validated emotion judgment task based on the Reading the Mind in the Eyes Test (RMET-R) ^3^. In this task, participants are asked to match grayscale images of human eyes to corresponding mental state labels. In autistic participants, intranasal OT administration has been shown to improve behavioral performance in this task^14^ and enhance social-related neural activations correlated with endogenous^45^ or peripheral OT levels^9^. Here, participants performed a revised version of this task, based on the study by Gordon et al. (2013), in which the original control condition (gender attribution) was changed to pictures of vehicles, which served as non-social control stimuli. This version allows us to test the social versus non-social effects of OT. Each trial began with a fixation cross, on which the participants were asked to focus their gaze. Then, an image appeared for one second, followed by a single word. Participants had to decide, using a button press, whether the word described the image. Overall, we presented 160 stimuli—20 different images in each category with four repetitions of each set. The order of the blocks, stimuli, and conditions was random and counterbalanced across participants (see Fig. 1).

Participants were placed in a supine position in the MEG apparatus, and stimuli were presented reflected on a mirror from a 17" screen located 60 cm above the participant's head using e-Prime software (version 2.0 professional, Psychological Software Tools, USA).

### Data analysis

#### Behavioral analysis

A generalized linear mixed model analysis was conducted for each participant using JAMOVI software^46^. The analysis was composed of two factors: study (OT/PL/TD) and condition (social/non-social). Accuracy (ACC) and reaction times (RTs, from correct trials only) served as dependent variables. A random intercept was included in the regression model adjusted by the clusters. For each participant in the ASD OT study, session trials (PL and OT) were clustered to establish a within-subject comparison in each analysis. Finally, we correlated individual parameters (age, WASI, and ADOS) with the effect of OT on each condition.

#### MEG preprocessing analysis

**Figure 2:**
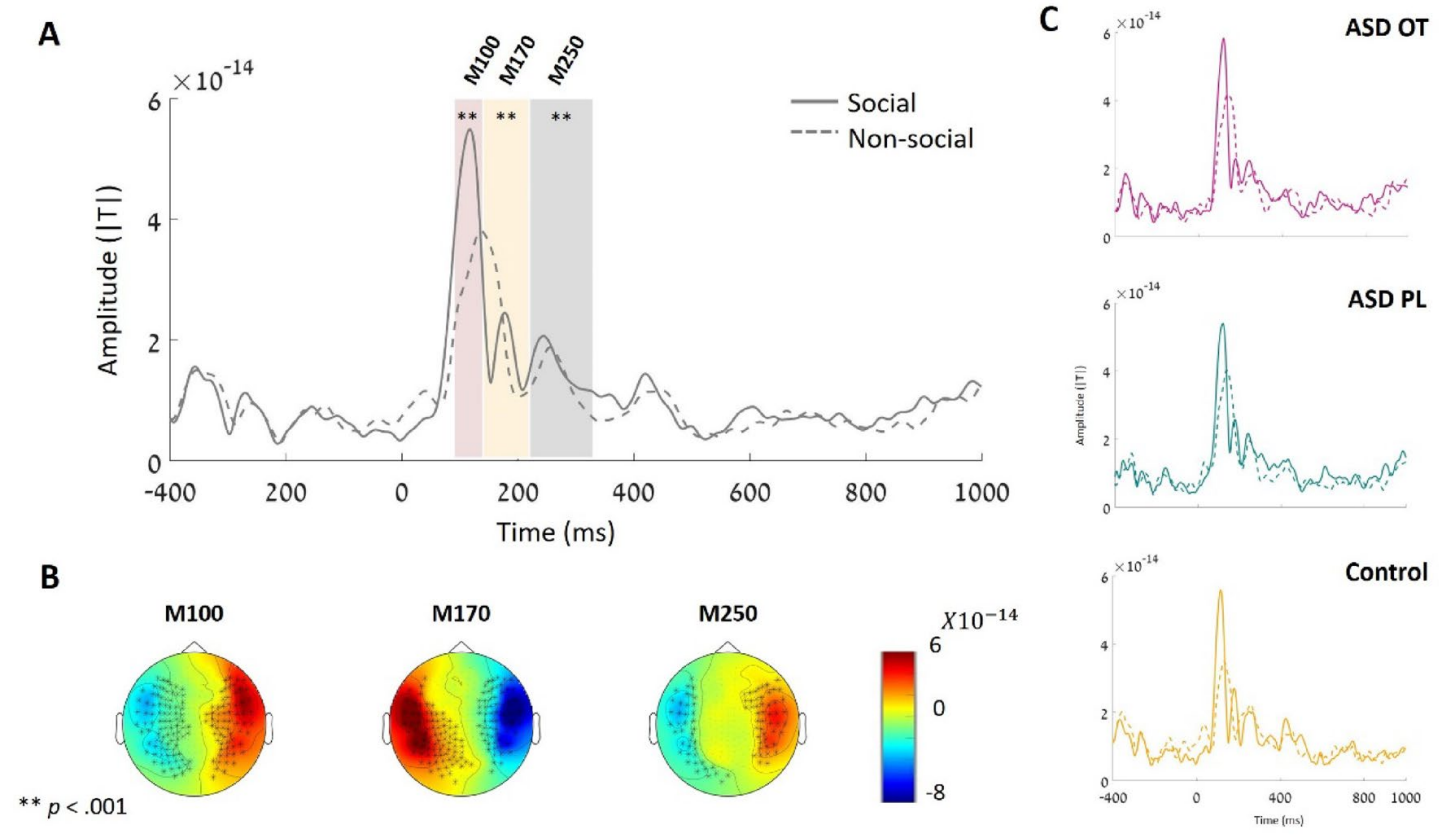
Evoked responses locked to social and non-social stimuli. (**A**) Root mean square (RMS) responses to social and non-social stimuli averaged for all experimental groups. (**B**) Scalp topographies showing the differences between social and non-social neural responses in each component (M100 – left, M170 -middle, M250 -right). Black dots represent MEG sensors with a significant difference in the average neural response between social and non-social stimuli. (**C**) RMS responses to social and non-social stimuli for each study group separately: ASD-OT (top panel), ASD-PL (middle panel), and TD (bottom panel).

A time-locked analysis was performed to inspect the neural dynamics in response to social stimuli compared to non-social stimuli at both the sensor and source levels. Three components, related to face processing, that are well known in the literature were selected in a preliminary experiment: M100, M170, and M250. The first, M100 (time: 90–140 ms), reflects primary visual processing and relates to social stimuli processing. M170 (time: 140–180 ms) relates specifically to face processing compared to other stimuli. Finally, we focused on rM250 (time: 220–330 ms), a component representing a neural response to a repetitive series of social stimuli, which was the nature of our paradigm. Time windows were selected based on previous studies in the field of face perception^32,47,48^. We verified, via manual inspection that the selected time windows included the peaks of the components in both the first and second levels (see Fig. 2 for the group-averaged signal). At both the sensor and source levels, we used the averaged amplitude of the neural activity in each time window to compare the conditions.

Brain activity was recorded using a whole-head 248-channel magnetometer array (Magnes 3600WH; 4-D Neuroimaging, San Diego, CA) inside a magnetically shielded room. Data were sampled online at 1017.23 Hz with a bandpass filter of 0.1–400 Hz. Five coils were attached to the head to record head positions and motions throughout the experiment. The head shape was digitized manually for subsequent source estimation. We used predesigned algorithms to remove heartbeat artifacts, line power (50 Hz and its harmonics), and excessive external noise^49^. Reference coils located above the head were used to remove environmental noise. Using the Fieldtrip toolbox for MATLAB^50^, data were then segmented into 1,400 ms. epochs: 400 ms. before the picture appeared and 1000 ms. following. We first removed channels with prolonged electrical abnormalities. After applying a high pass filter at 60 Hz, segments containing muscle artifacts were removed by visual inspection. Finally, all segments were filtered in the 1–30 Hz range with 10 s of padding. Independent component analysis (ICA) was applied (after resampling data to 300 Hz) to remove eye blinks, eye movements, and remaining heartbeats. A trial-by-trial inspection was conducted to reject unusual trials. Finally, all trials were time-locked for picture appearance and averaged separately for each condition^51^. Due to the complexity and size of the individual scanning data files All data is available upon request.

#### Event-related field (ERF) analysis

A time-locked analysis at the sensor level was performed to validate our task and the selected time windows (M100, 90–140 ms; M170, 140–180 ms, and M250, 220–230 ms). For each component, ERFs were calculated by averaging the individual neural responses across trials for social and non-social conditions separately. The inspection was conducted on the averaged evoked responses across all participants (TD, ASD under OT, ASD under PL). For each time window, paired t-tests were performed between sensors (threshold *α* = 0.05) and corrected for multiple comparisons using a cluster-based permutation procedure (1,000 permutations, statistic: the sum of the T-values within the cluster). In all three components, our analysis revealed significant differences in the amplitude of the locked neural response to social stimuli compared to non-social stimuli (M100: *p* < 0.001; M170: *p* < 0.001; M250: *p* < 0.001) (see Fig. 2), indicating that all of them showed the expected differentiation between social and non-social stimuli.

#### Source level analysis

Neural sources of the activation patterns were estimated with a linearly constrained minimum variance (LCMV) beamformer^52^ using custom-made analysis scripts. We fit a template MRI from the NIH pediatric database of young adult brains (ages 13–18.5 years)^53^ to the individual digitized head shapes using SPM12 (Wellcome Department of Imaging Neuroscience, University College London, London, UK). Next, we segmented the brain model into a canonical 1 cm grid and realigned it to the AAL atlas for subsequent ROI identification. We used all data covariance to obtain the common spatial filter, which was later applied separately to each condition. After estimating the source-level neural activity for each individual, we divided the statistical analysis into two steps.

### Effects of OT on the brain in autistic individuals

Our main objective was to investigate the effects of OT on early perceptual responses to faces in autistic adolescents. Using a whole-head analysis, we first examined the interaction between experimental conditions and sessions in the ASD OT study. For each voxel, we subtracted the brain activity in response to non-social trials from that which appeared in social trials for each group separately. We then compared OT and PL sessions using within-subject t-tests (corrected for multiple comparisons). This analysis was separately conducted for each time window. Finally, we investigated the main effect of OT by comparing the neural activity in OT and PL sessions above task conditions in each component of interest separately.

### Identifying social-related ROIs in the TD study and testing these regions for effects in the ASD OT study

The secondary objective of this study was to pinpoint social-related ROIs in this MEG task and to find significant clusters where higher levels of activation were observed for social cues than for non-social cues in TD individuals. Using repeated measures ANOVA, we first validated the differences in these ROIs between the TD study group and the ASD OT study groups (see supplementary information). Next, we examined the effects of OT on the ASD study only by comparing the neural activity of these ROIs between PL and OT sessions.

All mentioned source-level analyses in the source space were corrected for multiple comparisons using nonparametric cluster-based permutation t-tests (1,000 permutations, threshold *α* = 0.05, statistic: the sum of the t-values within the cluster), and the results were adjusted with the Bonferroni correction for multiple comparisons.

## Results

### OT increases early activation of frontal regions in autistic individuals in response to social stimuli.

**Figure 3:**
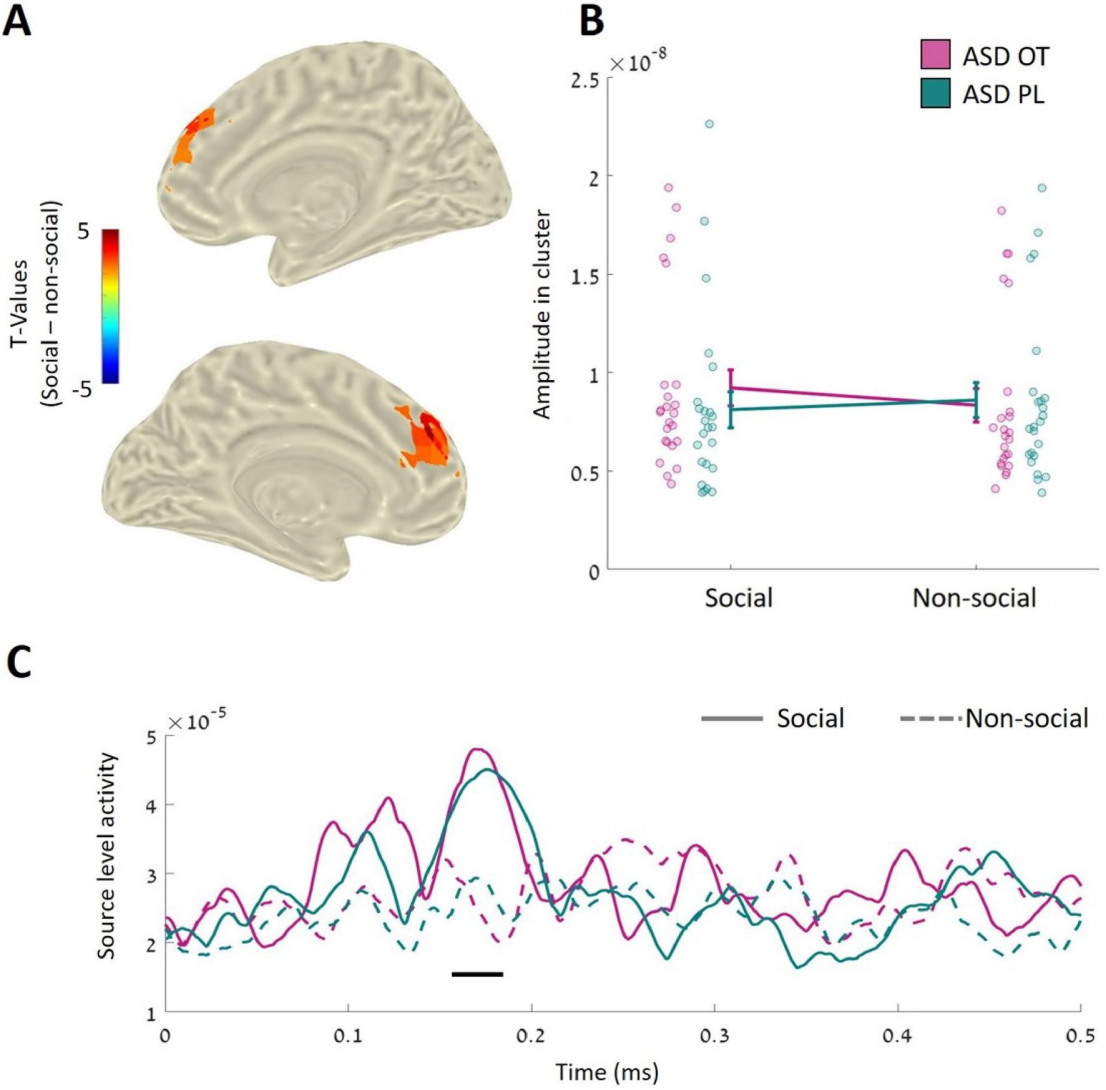
Source-level interaction between OT and PL sessions in the M170 time window. OT increased neural activation in medial frontal regions in response to social stimuli compared to non-social pictures. (**A**) Significant differences in neural activity between social and non-social stimuli were identified based on the t-values. (**B**) The average power of the significant cluster. Lines represent the average activity of the ASD group in each session. Dots represent individual data. (**C**) Source-level activity from a virtual sensor in the significant cluster. The horizontal black line represents the significant time window (*p* < .05, corrected for multiple comparisons). Straight lines represent neural activity in social trials, the dashed line represents activity in non-social trials. The color-coding is the same as in panel B.

For each component (M100, M170, and M250), the effects of OT in autistic individuals were located using a whole-brain LCMV beamformer analysis. The analysis revealed a significant interaction between the task condition and experimental session in superior and medial frontal regions (*p* < 0.05, corrected) during the M170 time window only (see Fig. 3 and Table 2). The post hoc analysis revealed that the difference between conditions was greater in OT sessions (*t*_(23)_ = 3.03, *p* = 0.006, Cohen's *d* = 0.62) than in PL sessions (*t*_(23)_ =  − 1.46, *p* = 0.16, Cohen's *d* =  − 0.29). For both M100 and M250, no significant interaction effects were observed (*p* = 0.6 and *p* = 0.83, respectively).

**Table 2:** Source-level interaction between OT and PL sessions in the M170 time window—size and location of the significant cluster.

Cluster Num	MNI coordinates (cm)			Anatomic region	% of cluster
	**x**	**y**	**z**		
1	0	4	3	L medial frontal gyrus	63
	1	5	4	R medial frontal gyrus	18.50
	− 2	6	3	L superior frontal gyrus	7.40
	− 2	5	3	L middle frontal gyrus	7.40
	2	5	4	R superior frontal gyrus	3.70

### OT increases early neural activation in the left frontal, occipital and temporal regions of autistic individuals.

**Figure 4:**
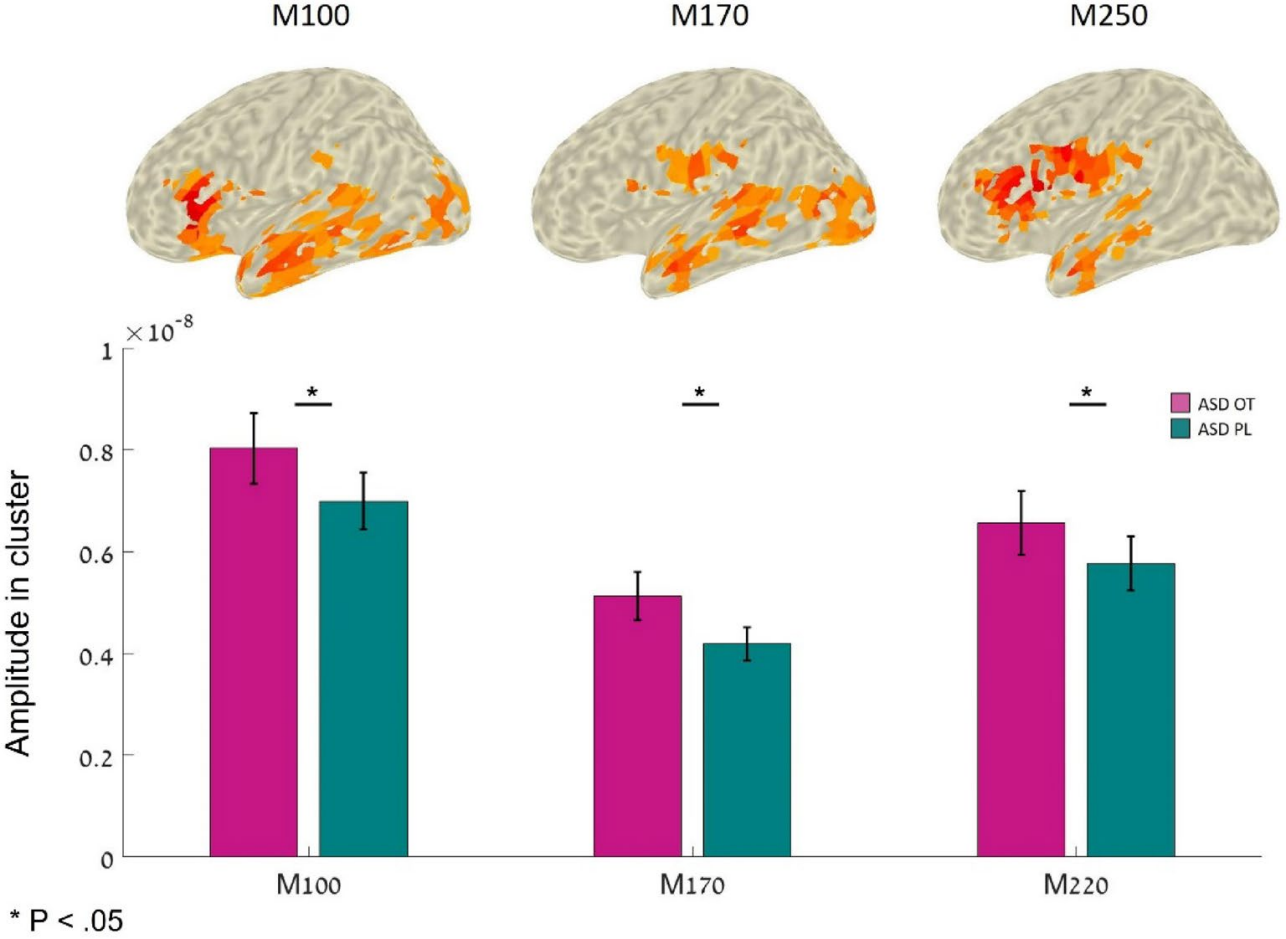
Main effect of OT is source-level. In general, compared to PL, OT increased neural activation in frontal, temporal, and occipital regions, regardless of the content (social/non-social) of the presented stimuli. Top panel–significant differences in each of the preselected components of interest between OT and PL sessions. Significant differences were observed in the left hemisphere only. Bottom panel–average power in the significant clusters for each group separately. Vertical lines represent mean values $\pm 1$ SE.

In addition to the interaction effect described above, a whole-brain analysis in the ASD OT study revealed a main effect of OT on all three-time windows (positive clusters: M100: *p* = 0.036; M170: *p* = 0.03; M250: *p* = 0.033, corrected for multiple comparisons). The pattern of the results was similar: significantly higher activation in frontal, occipital, and temporal regions in the left hemisphere only during the OT session than in the PL session (Fig. 4 and Table 3).

**Table 3:** Main effect of OT in all three components of interest—size and location of the significant clusters.

Cluster Num	time window	MNI coordinates (cm)			Anatomic region	Percent of cluster
		x	y	z		
1	M100 (90–140 ms)	− 6	0	− 2	L middle temporal gyrus	24.14
		− 6	− 1	− 3	L inferior temporal gyrus	18.97
		− 5	3	0	L inferior frontal gyrus, triangular part	12.07
		− 3	− 10	0	L middle occipital gyrus	8.62
		− 6	0	− 1	L superior temporal gyrus	6.90
		− 4	1	− 2	L superior temporal pole	6.90
		− 5	3	− 1	L orbitofrontal cortex	4.31
		− 6	1	1	L inferior operculum	3.45
		− 3	1	− 2	L insula	3.45
		− 5	− 7	− 1	L inferior occipital gyrus	3.45
		− 6	− 3	4	L supramarginal gyrus	2.59
		− 5	1	− 3	L middle temporal pole	2.59
		− 4	− 5	− 2	L fusiform	1.72
		− 5	4	2	L middle frontal gyrus	0.86
2	M170 (140–180 ms)	− 6	0	− 2	L middle temporal gyrus	31.96
		− 4	− 8	1	L middle occipital gyrus	16.49
		− 6	− 4	2	L superior temporal gyrus	10.31
		− 6	− 2	3	L postcentral gyrus	8.25
		− 6	− 4	3	L supramarginal gyrus	7.22
		− 2	− 10	− 1	L inferior occipital gyrus	5.15
		− 6	− 1	− 3	L inferior temporal gyrus	4.12
		− 6	1	3	L precentral	2.06
		− 6	1	2	L inferior operculum	2.06
		− 6	2	3	L inferior frontal gyrus, triangular part	2.06
		− 1	− 10	− 1	L calcarine sulcus	2.06
		− 6	− 4	4	L inferior parietal gyrus	2.06
		− 5	2	− 2	L superior temporal pole	2.06
		− 6	0	1	L rolandic operculum	1.03
		− 1	− 10	1	L superior occipital gyrus	1.03
		− 3	− 7	− 1	L fusiform	1.03
		− 5	1	− 3	L middle temporal pole	1.03
3	M250 (220–330 ms)	− 6	2	2	L inferior frontal gyrus, triangular part	15.79
		− 6	1	3	L precentral gyrus	14.91
		− 6	0	2	L postcentral gyrus	12.28
		− 6	0	− 3	L middle temporal gyrus	11.40
		− 6	− 3	2	L superior temporal gyrus	10.53
		− 5	4	2	L middle frontal gyrus	7.89
		− 6	1	2	L inferior operculum	6.14
		− 6	− 3	3	L supramarginal gyrus	5.26
		− 5	2	− 2	L superior temporal pole	3.51
		− 6	− 1	− 3	L inferior temporal gyrus	3.51
		− 3	3	1	L insula	2.63
		− 6	0	1	L rolandic operculum	1.75
		− 5	1	− 3	L middle temporal pole	1.75
		− 5	2	− 1	L orbitofrontal cortex	0.88
		− 6	− 4	4	L inferior parietal cortex	0.88
		− 6	− 1	1	L heschl gyrus	0.88

**Figure 5:**
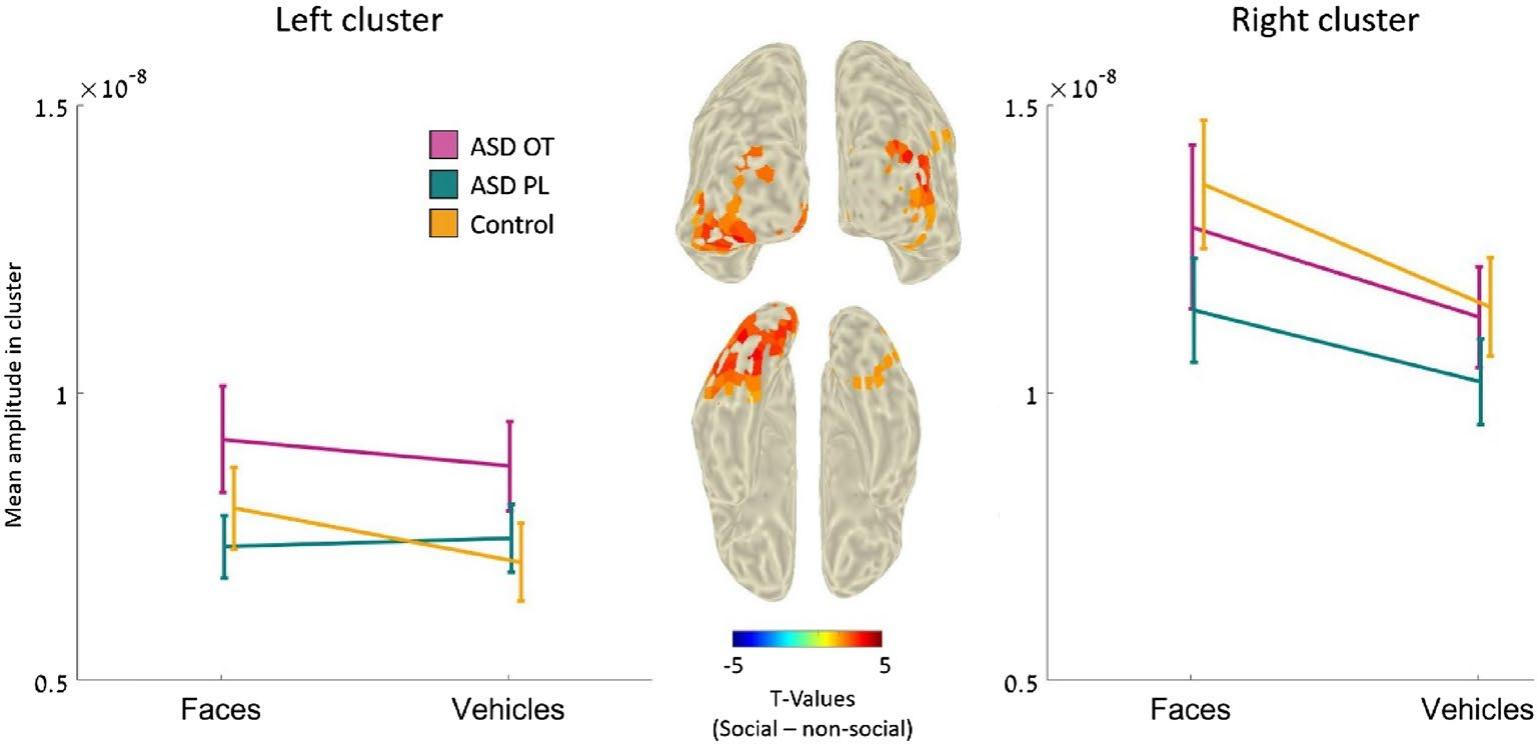
Neural activity in typical social-related ROIs of the experimental groups. These ROIs were pooled from the M170 time windows of the TD group. In this period, higher neural activity was observed in response to social stimuli than non-social stimuli. The middle section shows posterior and bottom views of social-related ROIs in the TD study. Color coding represents t-values. These regions represent neural regions that were more active in social trials than in non-social trials. The left and right panels show the average neural activity in these clusters (left and right, respectively) in each group separately.

The enhancement effect of OT in ASD was also noticeable in social-related ROIs and left and right clusters that were more active in response to social cues in the TD group (see Figure S2 and Table S1). Repeated measures ANOVA of these regions show a main effect of OT such that OT administration (compared to PL) increased neural activation in the left cluster but not in the right cluster in response to social and non-social cues (left: *F*_(1,23)_ = 4.12, p = 0.05, ${\eta }^{2}$=0.047; right: *F*_(1,23)_ = 2.7, *p* = 0.11, ${\eta }^{2}$=0.017). The results also reveal a main effect of the condition on the right cluster, as in this cluster, higher neural activation was observed in response to social stimuli (right: *F*_(1,23)_ = 6.83, *p* = 0.01, ${\eta }^{2}$=0.02; left: *F*_(1,23)_ = 2.9, *p* = 0.59, ${\eta }^{2}$ < 0.001) (see Fig. 5). The interaction was not significant in either of the clusters (left: *F*_(1,23)_ = 1.7, *p* = 0.02, ${\eta }^{2}$=0.002; right: *F*_(1,23)_ = 0.2, *p* = 0.65, ${\eta }^{2}$<0.001).

### Behavioral results

A generalized linear mixed model analysis was conducted for both accuracy and RTs. The ACC analysis included all trials in both studies (80 trials per condition), while RT analysis only included trials with correct responses (social: *M* = 62, SD = 5.69; non-social: *M* = 66.6, SD = 4.75).

The accuracy analysis revealed significant main effects of conditions (${\chi }^{2}$_(1)_ = 60.58, *p* < 0.001) and study groups (${\chi }^{2}$_(2)_ = 6.88*, p* < 0.05). The post hoc analysis revealed higher correct response rates under non-social conditions (*β* = 0.688, *z* = − 7.57 *p* < 0.001). We also observed significant differences between the TD study group and the ASD OT groups in PL sessions only, as TD participants presented higher accuracy rates (*β* = 0.788, *z* = − 2.57 *p* < 0.05). The interaction was not significant (${\chi }^{2}$_(2)_ = 0.21, *p* = 0.9).

**Figure 6:**
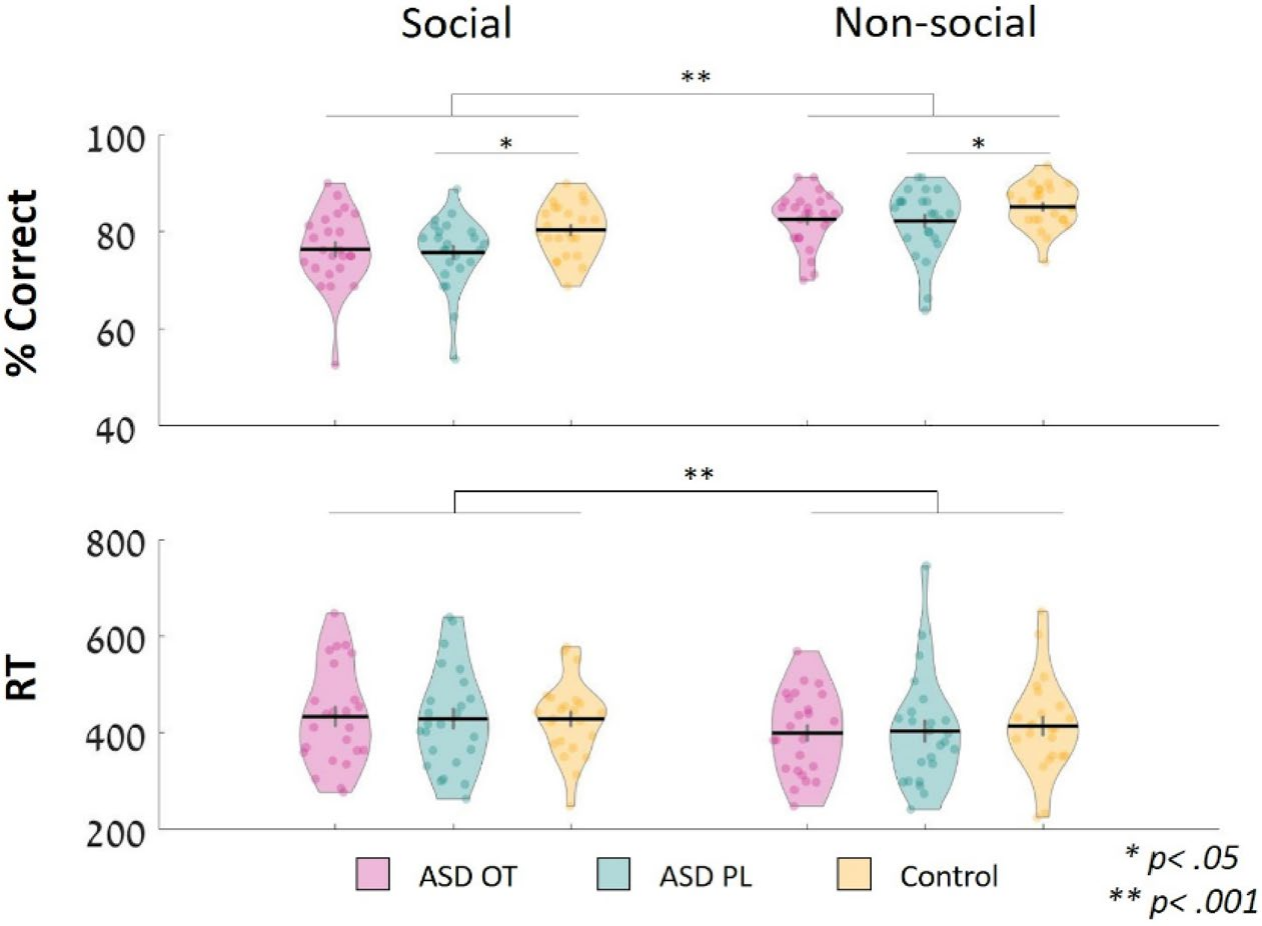
Behavioral results for accuracy rates and reaction times. After OT administration, the accuracy rates of the autistic individuals more closely resembled those of the TD study group. Analyses also revealed that all individuals presented higher ACC and lower RTs in the non-social trials. Accuracy (top panel) and RT (bottom panel) in response to social (left side) and non-social (right side) stimuli in the ASD-OT, ASD-PL, and control groups. Black lines represent the mean values of the groups. Vertical lines represent 1 SEM. Dots represent the individual data in the group.

The RT analysis also revealed a significant difference between conditions (${\chi }^{2}$_(1)_ = 16.97, *p* < 0.001), as RTs in non-social trials were shorter than those in social trials (*z* = 4.12, *p* < 0.001). No significant differences were observed for the study groups (${\chi }^{2}$_(2)_ = 0.76, *p* = 0.68) or interaction (${\chi }^{2}$_(1)_ = 1.17, *p* < 0.56) (see Fig. 6).

A significant positive correlation was identified between behavioral performance after OT administration and individual ADOS scores: the higher the score, the higher the differentiation between social and non-social accuracy rates in the OT (but not PL) session (*OT: r* = 0.46, *p* = 0.024; PL: *r* = − 0.15, *p* = 0.47, not corrected). No correlation was found between the OT effect and WASI score (*OT: r* = − 0.04, *p* = 0.85; PL: *r* =  − 0.1, *p* = 0.65, not corrected).

No correlation was observed between performance on the task and the neural effects of OT on the frontal and posterior regions (see SI for ROI definitions), either in the social (frontal: *r* = 0.08, *p* = 0.7; left posterior: *r* =  − 0.1, *p* = 0.65; right posterior: *r* =  − 0.14, *p* = 0.5) or non-social trials (frontal: *r* = 0.31, *p* = 0.13; left posterior: *r* =  − 0.24, *p* = 0.26; right posterior: *r* =  − 0.18, *p* = 0.38).

## Discussion

The current study examined the effects of OT administration on early attention toward social cues in autistic adolescents. By utilizing MEG imaging in combination with OT administration in autistic youth, we were able to identify a two-level mechanism by which OT affects perception. First, OT increased social-related neural activation in superior and medial frontal regions during the M170 time window. Second, OT increased neural activity in several social and non-social regions during the early phases of perception, regardless of the stimulus content. Below, we will elaborate on each pathway.

### First pathway: OT exerts distinct effects on social-related frontal regions

We first showed that OT modulates social perception in medial and frontal regions. These regions, which tend to be hypoactive in autistic individuals^54^, are crucial for social functions, such as mentalization^55^. This increased activation of frontal regions in response to social stimuli was observed only in the M170 time window, which is known to reflect cognitive processes of face recognition and identification^32^. Thus, consistent with other imaging studies showing higher socially related neural activation after OT administration in ASD^9,20,22,23^, our results suggest that OT enables more efficient and accurate processing of social cues and thus may improve the ability to interpret others' emotions by increasing activation in frontal regions. Gordon et al. (2013) suggested that OT represents the enhancement of signal-to-noise ratio (SNR) by increasing activity in social-related regions and reducing it in regions that are less relevant to the presented social perception. Thus, OT allowed individuals to be attuned more to the social stimuli in the task. Our results partly strengthen this hypothesis and expand it by showing that in frontal regions, OT increases the SNR even in early perceptual stages, such as the M170 time window.

Interestingly, the frontal complex was not identified as task-relevant in the TD study group (comparing social and non-social regions—see SI). We thus suggest that the unique effect of OT that is observed in the current study ASD group might reflect a compensation mechanism where acute administration of OT recruits the frontal regions, a region with expertise in social interpretation to enhance the overall social perception. This assumption needs a further examination of the connections between the frontal region and posterior areas and how they are influenced by OT.

### Second pathway: OT increased general early attention toward visual stimuli

Although several theories argue that OT mainly increases the salience of social cues^18,56^, our results show that during M100, M170, and M250, OT increased early neural activity in response to both social and non-social stimuli. One potential explanation for the differences between our results and the expected outcomes relates to our ability to target early temporal processes using MEG. The aforementioned theories mainly investigate the mechanism of OT through fMRI, which integrates early and late processing in a 2-s time frame, but the use of MEG allows us to focus on early stages in the processing timeline that reflects primary attention and perception time courses. A recent evolutionary-based theoretical perspective, allostatic theory^57^, suggests that OT facilitates early sensing, learning, and prediction to maintain stability in a changing environment. Our results regarding OT modulation are consistent with the allostatic theory, as we show that OT increased attentional preparation to the current stimulus, regardless of its social content, via an extensive amplification of neural activity in the left occipital, temporal, and parietal regions. These results are also consistent with several other studies highlighting the effects of OT on non-social functioning, such as decision making^58^, approach-avoidance behavior^59^, and clinical symptoms^60^^–^^62^. From this perspective, we suggest that one of the mechanisms by which OT improves the perception of social stimuli among autistic adolescents is, in fact, an early and widespread increase in the attentive capability to process the presented stimuli, regardless of their content.

In the current OT study, we did not find significant behavioral differences between OT and PL sessions. these results are in line with Gordon et al. (2013) paper which used a similar paradigm and did not find behavioral effects for OT. One possible explanation for these results is the lack of feedback throughout this task, which may impede an ability to improve and learn, which might be enhanced by OT. Another explanation is the fact that we modified the original fMRI task to fit the MEG setting,and reduced the number of labeling options. This might have resulted in a ceiling effect in the behavioral data, which could not be improved by OT administration. Despite the lack of behavioral differences, we believe that the reported null-effect do not diminish the significance of the observed neural effects of OT. In fact, these results highlight the need to validate the potential use of OT in ASD in combination with opportunities for feedback and learning^9,63^.

### Limitation

Although our study provides new insights into the effect of OT on the early processing of autistic adolescents, several limitations must be considered. The main limitation of our design is that we could not administer OT to individuals in the TD study due to the ethical guidelines for conducting OT research. We only examined the neural activity of TD adolescents who did not receive OT, which allowed us to identify “typical” ROIs and patterns of activity in response to our task and obtain new information regarding the differences between perception in TD adolescents and autistic individuals. However, to rule out confounds and assess generalizability, future research should aim to test which effects of OT are exclusive to ASD to ensure that the translation of OT use will be based on a deeper understanding of its effects and their specificity. This point is highly relevant, especially in light of theories that challenge the classic views of social deficits in autism by claiming that the observed communication differences point to an alternative perceptual channel that benefits the inter-autistic-group’s social communication ^7^. As such, despite the high clinical validation of the 'reading the mind in the eyes' test we used in order to gain an understanding of the influence of OT on social function in ASD, future studies should explore OT’s effects during real-life interactions between TD and autistic individuals and during social interactions between autistic individuals only.

Another limitation relates to the MIE task design, which needed to be adapted to the MEG setup. We changed the assignment and asked participants to agree if a single word represented the picture to reduce muscle artifacts due to eye movements. In the classic task^9,64,65^, children were asked to choose one word from four options. Although all individuals exhibited proper performance on the MIE in this study, the choice of an answer from multiple options might have enabled us to identify links between brain activity and behavior. We thus recommend that future studies collect more data in terms of time recording and task complexity.

Furthermore, several points should be mentioned regarding the characteristics of the participants in the current study. First, in the ASD study, the sample was quite homogenous and composed of autistic males with functional abilities. Future studies should further explore the effects of OT on a female sample and individuals with a variety of functional abilities to obtain an understanding of the full potential benefits of OT in autistic populations. Further to this point, since the TD study was used solely to identify social-related ROIs, participants were chosen according to their gender and age and were excluded based on medical information and parental reports only. We encourage future studies to match ASD individuals to TD individuals based on other critical individual properties such as IQ and social skills.

Finally, the experimental groups were composed of both left and right-handed participants. Former studies suggest that left-handed participants show less hemispheric lateralization in the FFA during face processing, compared to right-handed individuals^66,67^. Thus, it is possible that the effects of OT, in the pre-selected ROIs, may differ depending on individuals' hand preferences. This hypothesis should be examined with larger sample-sized studies thus allowing to control for handedness.

Nevertheless, the current study is unique in several ways, as it is the first to examine the effects of OT on autistic male youth using MEG; thus, it has important theoretical implications for a better understanding of the possible clinical benefits of OT in this population.

### Conclusion

In conclusion, our findings contribute to the accumulating evidence on the neural effects of OT in male autistic adolescents. By showing that OT modulates two early temporal neural pathways—both the widespread attentional pathway and the specific processes of the social cue pathway—our results extend the existing literature regarding the specification of effects of OT in autistic individuals and emphasize the importance of studying the effects of OT with high temporal resolution imaging techniques. In addition to the existing challenge of identifying individuals who will benefit the most from OT administration we highlight the potential of OT to enhance early processing of both social and non-social stimuli, in combination with behavioral treatments or opportunities for learning. This emphasis should be considered when devising optimal treatment methods in the future.

## Ethics statement

This study was carried out following the recommendations of the medical ethics committee of Israel and was approved by the Beer-Yaacov-Ness-Ziona Mental Health Center Ethics Board in accordance with the Helsinki declaration. Before the beginning of the sessions, participants' parents were provided an informed consent form (details on clinical registration can be found in www.clinicalTrial.gov, unique identifier: NCT05096676, registered as clinical trial October 27th, 2021).

## Supplementary Information

Supplementary Information.

## Data Availability

Due to the clinical features and the age of our sample as well as the sizes and nature of the imaging files, all data will be sent upon request. Please send an e-mail to the corresponding author for more details.
