# Supplementary material for: The dual neural effects of oxytocin in autistic youth: results from a randomized trial
Source: Sci Rep. 2022 Sep 29;12:16304. doi: 10.1038/s41598-022-19524-7 (PMC9523043; doi:10.1038/s41598-022-19524-7)
Supplement: Supplementary file 1 — Supplementary Information. [file 41598_2022_19524_MOESM1_ESM.docx]

**Supplementary information**

**Medication information**

Due to the high comorbidity between ASD and other neurological symptoms, medical treatment was not an excluded criterion. However, each individual's treatment was examined independently to evaluate the possible influence on the outcomes. A doctor accompanied the experiment, and each medicated participant was approved depending on the type of drug and the medical doses the individuals received.

The following are the names of the drugs whose use did not constitute a restrictive condition for participation in the trial.

- Fluoxetine (Flutine, Prizma)
- Strattera
- Risperdal (Risperidone)
- Adderall
- Aripiprazole
- Lisdexamfetamine (Vyvanse)
- Methylphenidate (Ritalin)
- Montelukast (Singulair)
- Gerdon
- Genotropin
- Escitalopram (Cipralex)


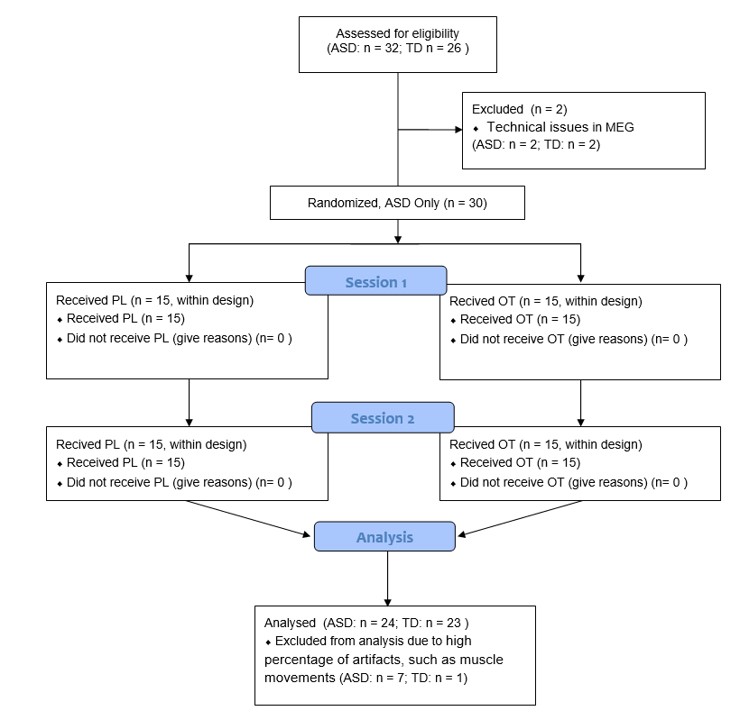


Figure S1: CONSORT flow diagram of the progress of the two groups in the ASD OT study through the phases of the experiment (enrolment, intervention allocation, and data analysis).

**Locating social-related ROIs in individuals in the TD study**

We extended the whole-brain approach to LCMV analyses to determine which regions in the TD study group were typically activated in response to social and non-social stimuli in the MIE. We aimed to locate specific ROIs that we later assessed for effects of OT in the ASD study group. We contrasted the neural activations of social and non-social trials in the TD study group using within-subject t-test analysis corrected for multiple comparisons to locate the specific neural regions with a greater response to social stimuli in TD adolescents.

Separate cluster-based permutation tests of each component in the TD study group revealed significant differences between conditions only in the M170 time window (140-180 ms). Activation was observed in two separate clusters (left hemisphere, positive cluster: *p* = .012, corrected; right hemisphere, positive cluster: *p* = .05, corrected) and mapped to occipital and temporal regions, including the occipital lobe, right and left fusiform gyrus, and calcarine sulcus (see figure S2 and Table S1). No significant differences were observed in M100 (*p* = .36) or M250 (*p* = .52).


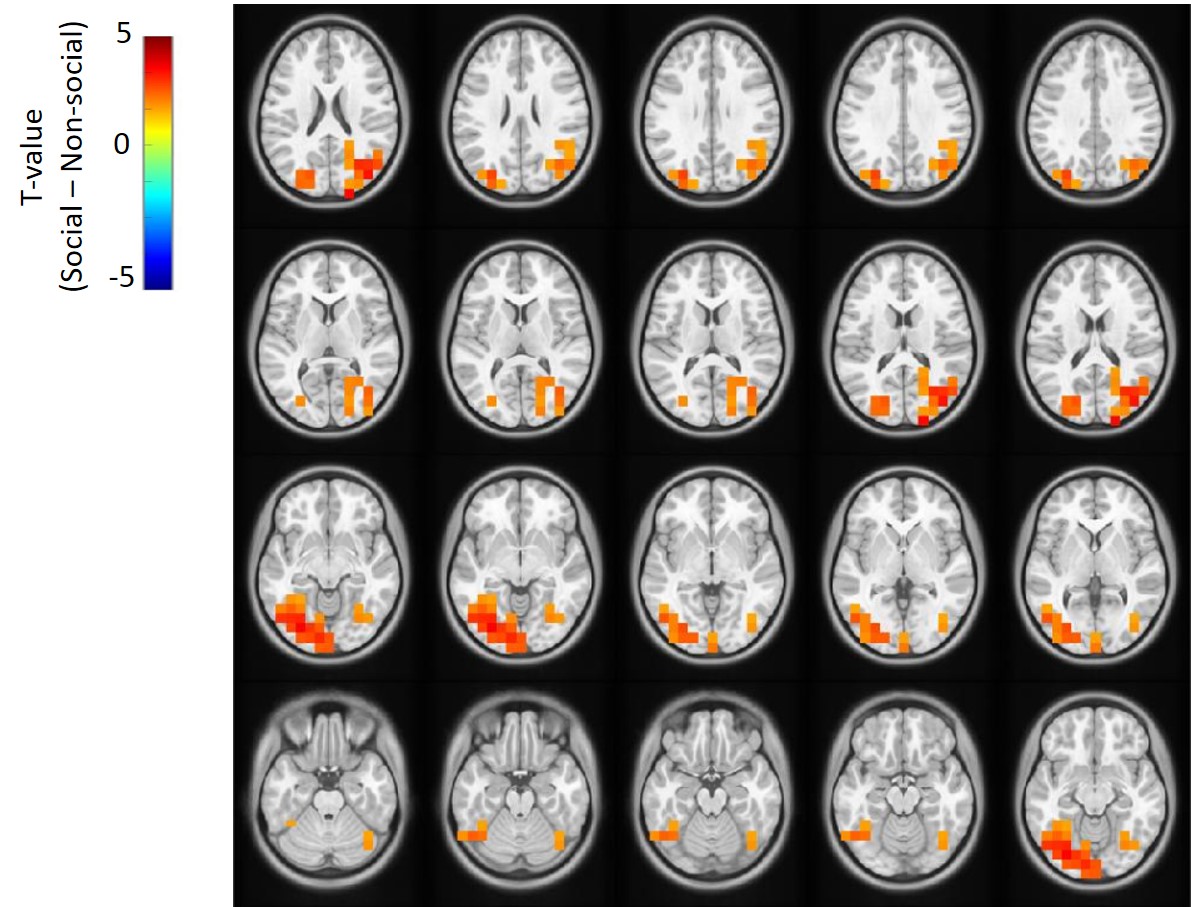


Figure S2: Social-related ROIs in the TD study group.

Table S1: Task relevant effects – social and non-social contrast in the TD study - size and location of the significant social related cluster.

| **Cluster Num.** | **MNI coordinates (mm)** | | | **Anatomic region** | **% of cluster** |
| --- | --- | --- | --- | --- | --- |
|  | x | y | z |  |  |
| 1 | -3 | -8 | 3.00 | L middle occipital gyrus | 30.95 |
|  | -3 | -8 | -1.00 | L inferior occipital gyrus | 16.67 |
|  | -1 | -9 | -1.00 | L Calcarine sulcus | 14.29 |
|  | -3 | -7 | -1.00 | L fusiform | 14.29 |
|  | -2 | -8 | -1.00 | L lingual cortex | 7.14 |
|  | -5 | -6 | -2.00 | L inferior temporal gyrus | 7.14 |
|  | -2 | -9 | 2.00 | L superior occipital gyrus | 4.76 |
|  | -5 | -7 | 0.00 | L middle temporal gyrus | 4.76 |
| 2 | 3 | -6 | 1.00 | R Calcarine sulcus | 11.76 |
|  | 2 | -9 | 1.00 | R cuneus | 8.82 |
|  | 2 | -10 | 2.00 | R superior occipital gyrus | 8.82 |
|  | 4 | -8 | 2.00 | R middle occipital gyrus | 29.41 |
|  | 4 | -7 | -1.00 | R inferior occipital gyrus | 2.94 |
|  | 3 | -7 | -1.00 | R fusiform | 11.76 |
|  | 5 | -7 | 3.00 | R angular gyrus | 11.76 |
|  | 2 | -5 | 2.00 | R precuneus | 2.94 |
|  | 4 | -7 | 2 | R middle temporal gyrus | 11.76 |

Next, we examined the differences in the neural responses of these ROIs between TD adolescents and autistic individuals (who participated in the OT study) during the N170 time window. We averaged the neural activity of the ROIs in the ASD OT study group during the PL session and compared it to the average response of the ROIs in the TD study group using repeated measures ANOVA (where 'group' was a between-subject factor and 'condition' was a within-subject factor).

Using repeated measures ANOVA with 'condition' as the within factor and 'group' as the between-subject factor, we first compared the neural activity of these ROIs in TD adolescents and autistic individuals treated with PL. A significant correlation was observed between ASD and TD groups in PL sessions in the left cluster only (left cluster: F_(1,44)_ = 8.5, p = 0.006, $\eta^{2}$= 0.008; right cluster: F_(1,44)_ = 1.18, p = 0.28, $\eta^{2}$= 0.003). A post hoc analysis using a t-test in the left cluster revealed that while a significant difference between conditions emerged in the TD study group (t_(21)_ = 4.12, p < .001, Cohen's d = 0.878), it was absent in the PL sessions (t_(23)_ = -0.52, p = .6, Cohen's d = -0.1).

**Brain-Behavior correlations**

We first examined the correlation between the effect of OT on frontal regions and the clinical assessment of participants’ abilities. No significant correlation was observed (WASI: social: r=- 0.004, *p*=0.98; non-social: r=- 0.02, *p*=.83; or ADOS social: r=- 0.166, *p*=0.44; non-social: r=- 0.1, *p*=.63).

We also examined the correlation between the neural effect of OT and behavioral performance on the task using two indices. We calculated the effects of OT by subtracting the neural activity and the behavioral performance in the PL session from that of the OT session for each condition separately. For the neural score, we used the average neural activation in three clusters: frontal regions, where an interaction effect was observed, and typical posterior ROIs, where we located the main effect of OT. Next, we correlated the neural and behavioral indices with each other. As mentioned in the main text, no interaction was observed for social and non-social cues.
